# Diapause characterisation and seasonality of *Aedes japonicus japonicus* (Diptera, Culicidae) in the northeast of France

**DOI:** 10.1051/parasite/2021045

**Published:** 2021-05-26

**Authors:** Eva Krupa, Nicolas Henon, Bruno Mathieu

**Affiliations:** 1 Université de Strasbourg, DIHP Dynamique des Interactions Hôte Pathogène UR 7292 67000 Strasbourg France

**Keywords:** *Aedes japonicus japonicus*, East Asian bush mosquito, Egg, Diapause, Morphology, France

## Abstract

The invasive mosquito *Aedes japonicus japonicus* (Theobald, 1901) settled in 2013 in the Alsace region, in the northeast of France. In this temperate area, some mosquito species use diapause to survive cold winter temperatures and thereby foster settlement and dispersal. This study reports diapause and its seasonality in a field population of *Ae. japonicus* in the northeast of France. For two years, eggs were collected from May to the beginning of November. They were most abundant in summer and became sparse in late October. Diapause eggs were determined by the presence of a fully developed embryo in unhatched eggs after repeated immersions. Our study showed effective diapause of *Ae. japonicus* in this part of France. At the start of the egg-laying period (week 20), we found up to 10% of eggs under diapause, and this rate reached 100% in October. The 50% cut-off of diapause incidence was determined by the end of summer, leading to an average calculated maternal critical photoperiod of 13 h 23 min. Interestingly, diapause was shown to occur in part of the eggs even at the earliest period of the two seasons, i.e. in May of each year. Even though we observed that the size of eggs was positively correlated with diapause incidence, morphology cannot be used as the unique predictive indicator of diapause status due to overlapping measurements between diapausing and non-diapausing eggs. This study provides new knowledge on diapause characterisation and invasive traits of *Ae. japonicus*.

## Abstract (fr)

*Aedes japonicus japonicus* (Theobald, 1901), un moustique invasif, s’est installé en 2013, en Alsace, dans le nord-est de la France. Dans une telle région tempérée, certaines espèces de moustiques ont recours à la diapause pour survivre aux températures froides de l’hiver, ce qui favorise donc l’établissement et la dispersion de ces espèces. Cette étude montre le phénomène de diapause et la saisonnalité d’une population sauvage d’*Ae. japonicus* dans le nord-est de la France. Pendant deux ans, les œufs ont été collectés de mai à début novembre. Ils étaient plus abondants durant l’été et plus rarement présents en octobre. Les œufs diapausants ont été déterminés par la présence d’un embryon entièrement développé dans les oeufs non éclos après des immersions répétées. Notre étude a mis en évidence une diapause effective d’*Ae. japonicus* dans le nord-est de la France. Au début de la période de ponte (semaine 20), jusqu’à 10 % des œufs a été trouvé à l’état de diapause et ce taux atteint 100 % en octobre. Un taux de 50 % de diapause a été atteint à la fin de l’été, amenant à une photopériode critique maternelle calculée de 13 heures 23 minutes en moyenne. De façon intéressante, il a été montré que la diapause se produisait chez une partie des œufs, et ce même dès le début des deux saisons, en mai de chaque année. Bien que nous ayons observé que les mesures des œufs étaient positivement corrélées avec le taux de diapause, la morphologie des œufs seule ne peut pas être utilisée comme unique indicateur de prédiction du statut de diapause, à cause d’un chevauchement des mesures entre les œufs diapausants et non diapausants. Cette étude offre de nouvelles connaissances sur les caractéristiques de la diapause et sur les traits d’invasion d’*Ae. japonicus*.

## Introduction

The East Asian bush mosquito, *Aedes japonicus japonicus* (Theobald, 1901), is native to eastern Asian countries like Japan, Korea and China [48] and has colonised the world mainly through trade in used tyres [32]. This invasive mosquito began spreading in the United States in 1998 [1] and started to colonise Europe two years later, in 2000 [39]. Nowadays, in Europe, the species is found in France [39], Belgium [54], Switzerland [40], Germany [5, 20, 55], Austria [44], Slovenia [44], the Netherlands [17], Luxembourg, Liechtenstein, Spain, Croatia, Hungary, Italy [36], Bosnia and Herzegovina, and Serbia [19]. The East Asian Bush mosquito has therefore been able to colonise a vast area and various latitudes in only 20 years. This highlights its great ability to adapt and to spread over a large geographical area under different meteorological conditions. It is likely that the subspecies *Aedes japonicus japonicus* is the only one present in Europe, called hereafter *Aedes japonicus*.

In France, *Ae. japonicus* was detected in 2000 [39] and settled in the northeastern part of the country in 2013 [23]. Prior to invasion by *Ae. japonicus,* mainland France had already faced invasive mosquitoes such as *Aedes albopictus* (Skuse, 1894), which is now considered the most invasive mosquito species in the world [50] and is well established in Europe [30]. The so-called tiger mosquito has been established in France since 2004 [10, 38] and was first detected in the northeastern part of the country in 2014 [24]. Both species, *Ae. albopictus* and *Ae. japonicus*, are now well settled in the northeast of France.

The invasive species *Ae. japonicus* is widespread and can feed on a wide range of hosts from birds to various mammals, including humans [41]. In addition, *Ae. japonicus* is a potential vector of several viruses such as chikungunya virus, dengue virus, Japanese encephalitis virus, Saint Louis encephalitis virus, and West Nile virus [29]. *Aedes japonicus* has colonised a wide range of areas and has a preference for forest edges [31]. Even though it is described as a forest species [27], it can be found in open areas. Tree holes and rock pools are its main natural larval habitats [1, 3, 43, 48, 52]. Artificial breeding sites like plastic buckets or rain barrels are regularly colonised [4, 21, 47, 52]. This species is more frequently found in rural than in urban areas: tyres in rural areas are more frequently stored outside and act as suitable breeding sites [4]. Cemeteries are also suitable places since larvae can develop in small water containers found around the tombs [5]. Artificial containers such as tyre casings, catch basins, large containers or surface water pools have also been mentioned as appropriate oviposition places [1, 6, 21], especially when they are a dark shade or colour [3, 4]. Thus, suitable places for this species are good vegetation cover, with available natural and artificial breeding sites and the presence of potential hosts like mammals, birds and humans in the surroundings.

Each mosquito species has its own phenology, which is mainly dependent on environmental variables like temperature or the availability of suitable water bodies [12]. The presence of *Ae. japonicus* adults was observed to be negatively correlated to temperature [4] and this mosquito is more active in spring and later, in autumn [21]. Its larvae also show tolerance to cool water temperatures, but a recent study observed no fourth instar larvae below 10 °C [8].

Culicids have developed various strategies at all the biological stages to survive through cold winters in temperate regions [11, 22]. One of these strategies is dormancy: described as an interruption of metabolic activity, it is a key parameter in maintaining natural populations [11].

Dormancy can be divided into quiescence or diapause. Quiescence is likely to cease when favourable environmental conditions return [11]. On the other hand, diapause is complex and is generally controlled by genetic or hormonal factors, meaning that it is seasonal and persists over time. Diapause is consequently a particular form of dormancy and constitutes an advantage for species dispersal and winter survival in colonised areas [49].

When temperatures decline in winter, *Ae. japonicus* larvae or eggs can survive in their areas of origin and colonised regions [3, 9, 48]. However, the life stages undergone by this species in winter seem to vary according to abiotic conditions and need further clarification in our study area. When eggs are in the overwintering stage, it is unclear whether their hatching inability is due to quiescence or diapause. Furthermore, even though it is highly suspected, diapause has not clearly been demonstrated in *Ae. japonicus*. The photoperiod is likely to be a factor that induces diapause [7], and a study showed that female adults of this species are photosensitive [7]. This would support the hypothesis that *Ae. japonicus* eggs are capable of diapausing. In addition, the shell structure of *Ae. japonicus* eggs shows desiccant resistance [54], which would also be in favour of this type of dormancy. Despite these possibilities, and as opposed to *Ae. albopictus*, it has not been shown whether *Ae. japonicus* eggs enter diapause. Even though higher temperatures can limit its spread [21], the Asian bush mosquito is a highly invasive vector species requiring surveillance. Characterising seasonality and diapause in *Ae. japonicus* is key to better understanding the colonisation process and spread of this invasive vector species, since these factors can increase the efficiency of control methods.

In this study, we describe diapause incidence and the seasonality of *Ae. japonicus* in the northeast of France. We investigated the overwintering of *Ae. japonicus* and determined which parameters influence diapause in eggs. To this end, we (i) described the morphology and viability of *Ae. japonicus* eggs throughout the year, (ii) characterised diapause in the late season, and (iii) predicted diapause in eggs with the help of unsophisticated and simple parameters.

## Methods

### Studied field area

**Figure 1:**
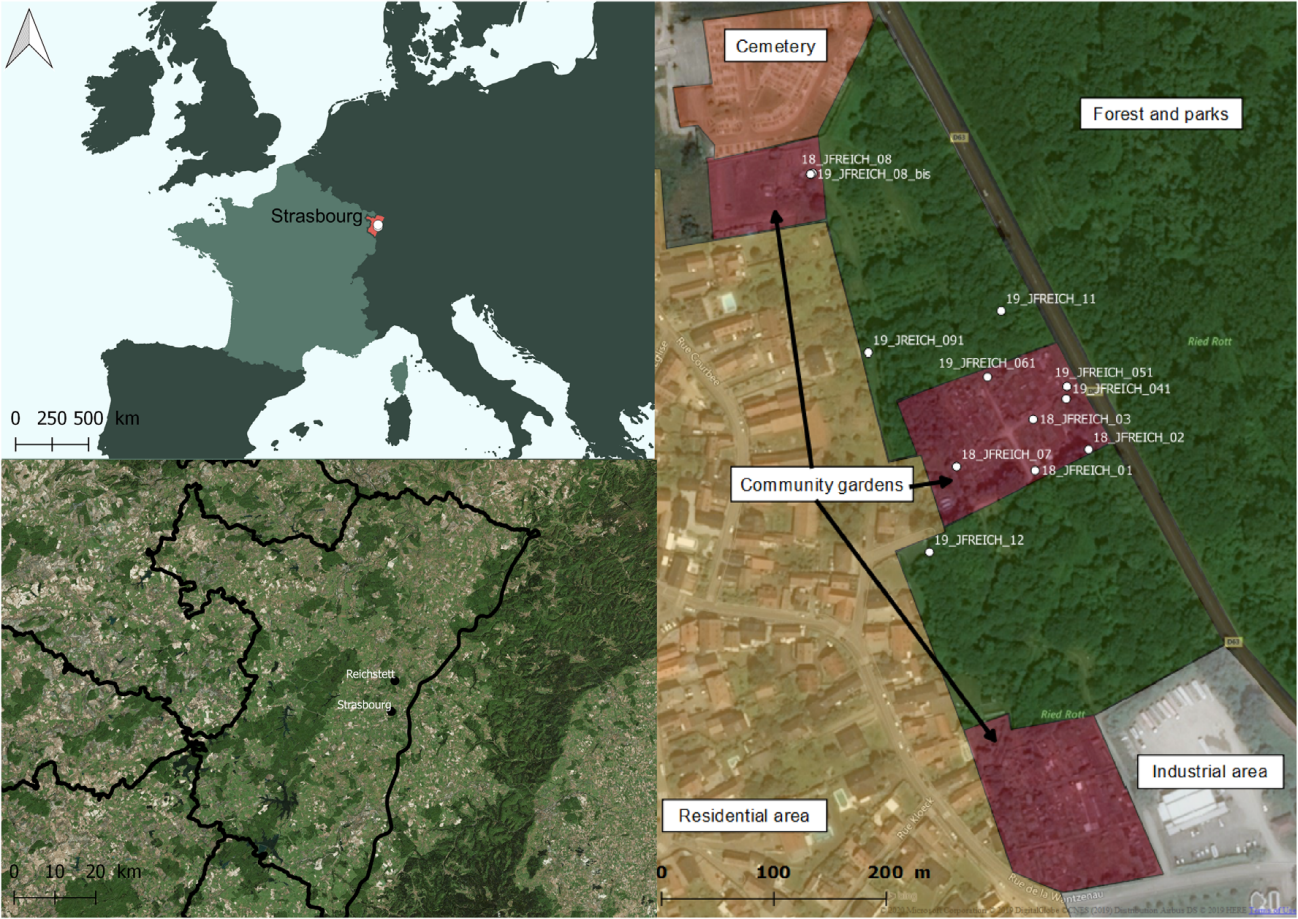
Field area of interest in Reichstett, France. Relative locations of Strasbourg and Reichstett in the Bas-Rhin Department (in red). On the right, details on land uses of the studied area and ovitraps position are given. Industrial areas (grey) are mainly made of concrete with no or few breeding sites. Residential areas (yellow) represent the main land use with individual houses and private gardens. The Reichstett cemetery (orange) is located on the north border of the city and provides flower pots and saucers or other small containers which serve as breeding sites. Three community gardens (red–violet) are inside the field area of interest. Patches with garden sheds and water storage are delimited by low height fences. Parks with planted trees or smaller natural forest areas (green) separate community gardens. A larger natural forest is located beyond the departmental road. Street map background is under ^©^OpenStreetMap & contributors.

Eggs were collected in the municipality of Reichstett, around 7 km north of Strasbourg (48.64 N, 7.75 E) (Fig. 1), where (i) a great number of *Ae. japonicus* eggs were repeatedly collected prior to this study, and (ii) no *Ae. albopictus* was present. The study area was approximately 82,000 m^2^ in size and delimited by roads and fences of private gardens. The composition of the Reichstett study site can be roughly divided into 1.7 ha of community garden (21.5%) and 6.5 ha of forest (78.5%), both areas being suitable habitats for this species. Supplementary eggs were collected during local monitoring in the Bas-Rhin Department in the northeast of France to follow the general egg pattern abundance (see Sect. Pattern of egg abundance in the field and diapause incidence related to environmental clues).

### Data collection and identification

Eggs were collected every two weeks by ovitraps. A trap consisted of a black bucket filled with 1.5 L of water and a piece of extruded polystyrene which served as an oviposition substrate. In order to increase the attractiveness of the ovitraps, local plant litter was added to the water. In the laboratory, eggs were counted under a Nikon SMZ1270 binocular magnifier (Nikon Corporation, Tokyo, Japan) and morphologically identified to species level using a Zeiss Standard 25 microscope (Zeiss, Oberkochen, Germany) and the egg description of *Ae. japonicus* by Haddow et al. [16]. Hatched larvae were identified at the fourth instar using the studies of Farajollahi and Price [13], and Tanaka et al. [48] while adult stages were confirmed by the description of Tanaka et al. [48]. The hatched, dried and damaged eggs collected were discarded prior to analysis.

### Egg collection

Weeks referred to the calendar week (ISO standard 8601 week date). A total of 12 ovitraps were set up in the Reichstett area and collected every two weeks from weeks 10 to 32 (6 March – 7 August). Furthermore, to increase details on diapause incidence in the late season, the sampling effort was led weekly from weeks 32 to 46 (7 August – 12 November) in 2019. In 2020, the collection was performed on a weekly basis during the whole sampling season from weeks 10 to 44 (2 March – 28 October). In addition, the general seasonality pattern of eggs was investigated from 158 to 98 ovitraps set up in the Bas-Rhin Department in 2019 and 2020, respectively. As part of the regional surveillance network for invasive mosquitos, they were collected every two weeks, from weeks 19 to 47 (6 May – 19 November) in 2019 and from weeks 25 to 41 (15 June – 10 October) in 2020.

### Morphological measurements

All the eggs were measured when fewer than 20 eggs were collected in the oviposition substrate. Otherwise, a 10% subsample of eggs was visually selected for each oviposition substrate (see Additional File 1, Table 1 for sampling size).

Each egg was prepared on a microscopic slide using a piece of double-sided adhesive tape. The eggs were observed under a Zeiss Standard 25 microscope equipped with a Nikon DS-Fi3 digital camera (Nikon Corporation) and an additional LED light source. The length and width of each egg were measured in μm with NIS elements software version 4.6 from Nikon Corporation. Each measure was taken three times to avoid observation bias [25]. Width was measured approximately at one third of the anterior part of the egg, which is both the widest part of the egg and the part where the egg shell breaks when the larvae hatch. Egg volume in ×10^−3^ mm^3^ was calculated using the following formula [2, 53]:

$$\mathrm{Volume}=\frac{1}{6}\times \pi \times \mathrm{length}\times {\mathrm{width}}^{2}.$$

### Hatching success, viability and diapause incidence in eggs

**Figure 2:**
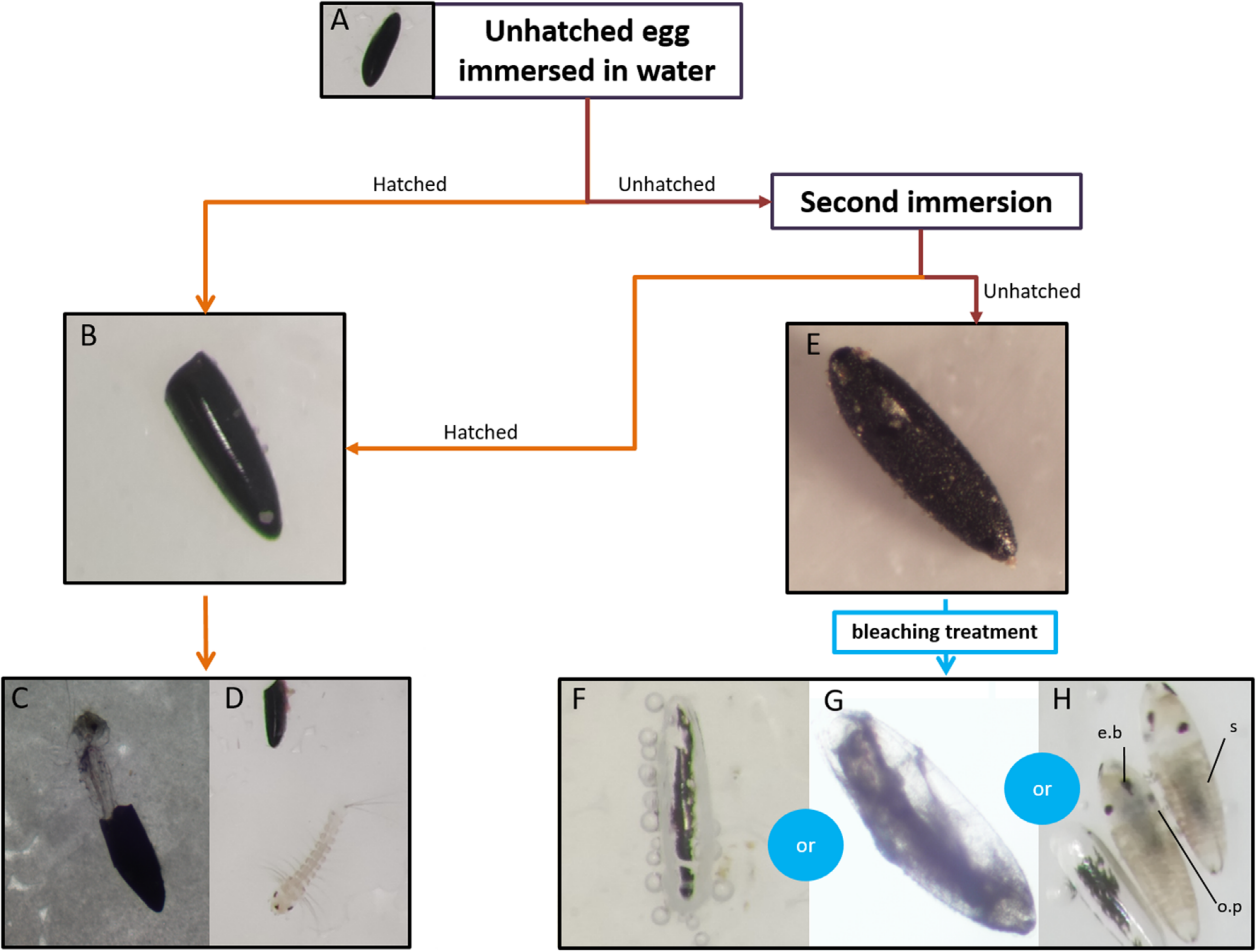
Assessment of hatching success, mortality rate and diapause incidence. Eggs (A) are immersed in tap water twice. After this hatching test, eggs can either hatch (orange arrows – B), resulting in a larva (C and D) out of the shell, or remain unhatched (red arrows – E), meaning the egg has not opened. Afterwards, unhatched eggs are bleached (blue arrows). In this case, unhatched eggs can either be unfertilised (F), partially embryonated (G) or contain a fully developed embryo (H). Full embryogenesis is achieved when complete segmentation (s), pigmented ocelli (o.p) and egg burster (e.b) are observed without deformity.

The study design made it possible to distinguish quiescence from diapause. From every ovitrap collected, 10–30 eggs were visually selected and submitted to the hatching procedure described in Figure 2. Repeated immersion of the eggs led to their hatching if they were under a state of quiescence. Conversely, eggs under diapause would remain unhatched until the end of this protocol. In order to determine hatching success, the batches of eggs (Fig. 2A) were immersed in 60 mL of tap water at room temperature for 24 h. After allowing the eggs to dry for 24 h at 22 ± 1 °C and 70% relative humidity, unhatched eggs were subjected to a second immersion period of 7 days. Hatched eggs (Fig. 2B) and larvae (Figs. 2C and 2D) were therefore counted to determine the hatching success rate at 24 h after the first immersion and at 24 h, 48 h and 7 days after the second immersion.

At the end of this procedure, unhatched eggs were dried for 24 h at room temperature and observed under a Nikon SMZ1270 stereomicroscope (Nikon Corporation). The unhatched eggs from the step illustrated in Figure 2E were bleached with Trpiš solution [51] for 10–20 min at room temperature. This bleaching treatment allowed us to observe the state of the embryos through the egg shells. Thus, the status of the unhatched eggs could be determined as follows: unfertilised (Fig. 2F), partially embryonated (Fig. 2G) or with a fully developed embryo (Fig. 2H). Unfertilised eggs had no visible content within the clear egg shell. Partially embryonated egg exhibited either a white unshaped mass or a partially formed embryo, according the following criteria: partial segmentation, ocelli partially formed or missing, egg burster missing. Full embryogenesis was observed when complete segmentation, pigmented ocelli and egg burster were visible without deformity. Eggs with a fully developed embryo are therefore referred to as embryonated unhatched eggs.

Therefore, hatching success, viability, mortality rate and diapause incidence were, respectively, calculated as follows:

$$\mathrm{Hatching}\mathrm{success}\%=\frac{\mathrm{Hatched}\mathrm{eggs}}{\mathrm{Embryonated}\mathrm{unhatched}\mathrm{eggs}+\mathrm{Hatched}\mathrm{eggs}}\times 100,$$

$$\mathrm{Viability}\mathrm{rate}\%=\frac{\mathrm{Embryonated}\mathrm{unhatched}\mathrm{eggs}+\mathrm{Hatched}\mathrm{eggs}}{\mathrm{All}\mathrm{eggs}}\times 100,$$

$$\mathrm{Mortality}\mathrm{rate}\%=\frac{\mathrm{Partially}\mathrm{embryonated}\mathrm{unhatched}\mathrm{eggs}+\mathrm{Unfertilised}\mathrm{unhatched}\mathrm{eggs}}{\mathrm{All}\mathrm{eggs}}\times 100,$$

$$\mathrm{Diapause}\mathrm{incidence}\%=\frac{\mathrm{Embryonated}\mathrm{unhatched}\mathrm{eggs}}{\mathrm{Embryonated}\mathrm{unhatched}\mathrm{eggs}+\mathrm{Hatched}\mathrm{eggs}}\times 100.$$

Diapause incidence can also be determined as:

$$\mathrm{Diapause}\mathrm{incidence}\%=100-\mathrm{Hatching}\mathrm{success}\left(\%\right).$$

### Predictive model of diapause incidence in eggs

In order to categorise eggs either as under diapause or not in the late season, a predictive model was proposed. Only viable eggs were considered for analysis. Eggs were batched per week of collection. A logistic model was applied to evaluate the probability of diapause in eggs, in which success was defined as an egg under diapause and failure as an egg not under diapause. The Akaike information criterion (AIC) method was used to select the week of collection and the width of an egg as the two best parameters to be implemented in the model. Thus, the logistic model was expressed as the following equation (1):

(1) $$\log\frac{p}{1-p}=\beta_{0}+\beta_{1}x_{1}+\beta_{2}x_{2}+\beta_{3}x_{1}x_{2}+\sum_{i=1}^{3}\varepsilon_{i}.$$

In this equation, *p* is the probability of the egg being under diapause, *β*_0_ the intercept, *β*_1_ the regression coefficient associated with *x*_1_, the explicative temporal parameter “week of collection”, *β*_2_ the regression coefficient associated with *x*_2_ which is the explicative morphological parameter “width of egg”, and *β*_3_ the regression coefficient of the interaction between *x*_1_ and *x*_2_. *ε*_*i*_ were associated errors.

In order to evaluate the link between diapause incidence and egg morphology, grouping was performed. The 50% diapause incidence cut-off was observed in week *n*, the mix of diapausing and non-diapausing eggs sampled from weeks *n* − 1 to *n* + 1 were gathered into a first group labelled M. The eggs collected earlier than week *n* − 1 were grouped as non-diapausing eggs (group ND) while the eggs sampled later than week *n* + 1 were grouped as diapausing eggs (group D). The proposed model was adjusted without group M as it contained a mix of diapausing and non-diapausing eggs.

### Meteorological data and photoperiod

Meteorological data were provided by meteofrance.com (Météo-France, Saint-Mandé, France) and came from the closest station in Strasbourg Entzheim (48.55 N 7.63 E) located 14 km away from the study site. The following meteorological data were collected: average minimal temperature per week (*avT*_min_), average maximal temperature per week (*avT*_max_), minimal value of minimal temperature per week (min*T*_min_), minimal value of maximal temperature per week (*minT*_max_), maximal value of minimal temperature per week (*maxT*_min_), and maximal value of maximal temperature per week (*maxT*_max_). Temperatures are expressed in degrees Celsius. Sunshine is expressed in hours:minutes and rainfall in mm. Sunshine and rainfall were also averaged for each calendar week resulting in average sunshine per week (*av*Sun) and average rainfall per week (*av*Rain), respectively. These two parameters were also summed up after seven consecutive days as weekly cumulative sunshine (*cumul*Sun) and weekly cumulative rainfall (*cumul*Rain).

The daily sunrise and sunset time in Strasbourg were used to define day length, i.e. daily photoperiod, which was subsequently expressed as a weekly average (*av*Photoperiod).

### Statistical analysis

All statistical analyses were performed with R language [34]. Two-tailed tests were performed with error type I *α* = 5%. Mean and median results were expressed as mean ± standard deviation (SD) or as median interquartile range (IQR).

To observe the pattern in egg laying, eggs from each ovitrap in the Reichstett area were cumulated per two weeks.

The eggs of week 45 in the year 2019 collected in Reichstett were discarded for both morphological and diapause analysis due to a low sample size (*n* = 5, see Additional File 1, Table 1 for sampling size). Diapause incidence was studied during summer and autumn, from weeks 26 to 44, each year.

Relationships between week of collection, number of eggs, diapause incidence, morphological measurements of eggs (length, width and volume) and meteorological parameters (*avT*_min_, *avT*_max_, *minT*_min_, *minT*_max_, *maxT*_min_, *maxT*_max_, *av*Sun, *av*Rain, *cumul*Sun, *cumul*Rain, *av*Photoperiod) were analysed through principal component analysis (PCA). Threshold was set at 75% of explained variance, and Spearman rank order correlations were calculated between these parameters.

Linear regressions were assumed between week of collection and width of the eggs as well as between week of collection and volume of the eggs. Adjusted *R*^2^ was used to evaluate model fitting. Linear regressions between weeks of collection and morphological measures of eggs were performed. Linear regression was expressed as the following equation (2):

(2) $$y=\beta_{0}+\beta_{1}x_{1}+\varepsilon_{1}.$$

In this equation, *y* is the morphological parameter studied (either the volume or the width of the eggs), *β*_0_ the intercept, *β*_1_ the regression coefficient, *x*_1_ the explicative parameter “week” and *ε*_1_ the associated errors.

Eggs were grouped according to the week of collection and the critical photoperiod in which diapause incidence reached 50% in eggs. As data were not normally distributed (Shapiro–Wilk test, *P* = 0.009), the Kruskal–Wallis test was performed in order to support the hypothesis of arbitrary groups being a homogeneous group. The one-tailed Wilcoxon rank sum test was performed to analyse the morphology between diapausing and non-diapausing eggs.

The maps were made with QGIS Las Palmas 2.18.23 [33].

## Results

### Pattern of egg abundance in the field and diapause incidence related to environmental clues

**Figure 3:**
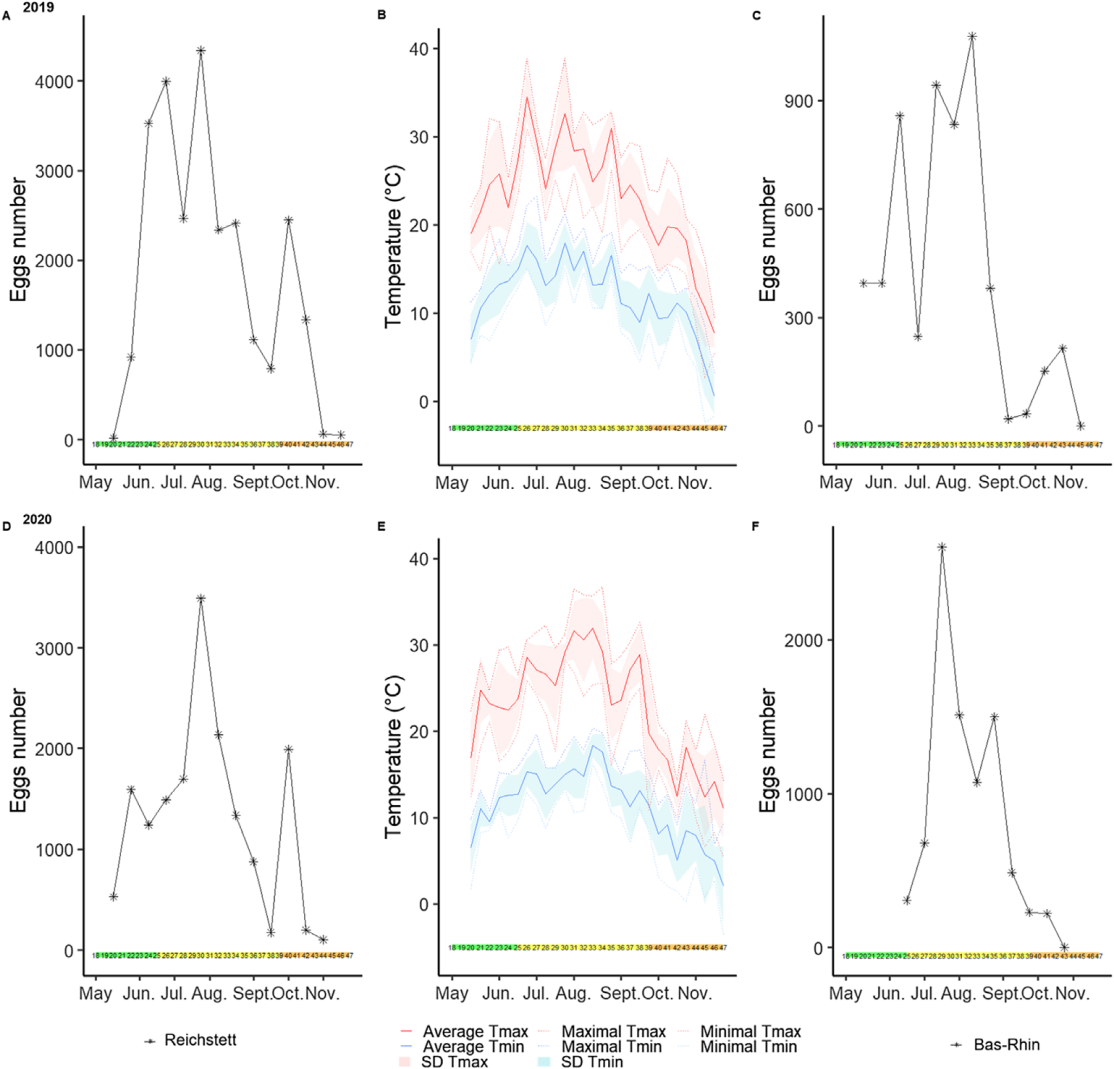
*Aedes japonicus* egg seasonality regarding environmental temperatures from May to November 2019 and 2020. Corresponding months and seasons are shown with coloured horizontal bars (green = spring, yellow = summer, orange = autumn). (A) 2019, egg seasonality in the Reichstett study site; (B) 2019, weekly temperatures are shown. The average maximal temperature per week is shown with a solid red line with the standard deviation shown in filled light red. Dotted red lines correspond to the maximal and minimal highest temperatures observed each week. Average minimal temperature is shown with a solid blue line with standard deviation in filled blue. Minimal and maximal colder temperatures are shown with dotted blue lines. (C) 2019, egg proportion from the Bas-Rhin Department is also shown with a solid black line with stars. (D) 2020, egg seasonality in the Reichstett study site; (E) 2020, weekly temperatures; (F) 2020 egg seasonality in the Bas-Rhin administrative unit.

In 2019, within the studied area, the 12 ovitraps sampled collected 25,809 eggs of *Ae. japonicus* from week 20 (mid-May – 16 eggs) to week 45 (first week of November – 51 eggs). *Aedes japonicus* eggs were detected in Reichstett over 25 consecutive weeks and the number of collected eggs ranged from 16 to 4339 eggs with an average of 1844 ± 1454 eggs per two-weeks. In 2020, the 12 ovitraps sampled 16,859 eggs from week 20 (mid-May – 529 eggs) to week 44 (end of October – 103 eggs), meaning that the East Asian bush mosquito was detected for 24 consecutive weeks. In 2020, the number of collected eggs ranged from 76 to 3491 eggs with an average of 1297 ± 955 eggs per two-weeks (Table 1). While most of the eggs were collected between June and August during both years (Fig. 3), the highest numbers of samples were collected in week 26 in late June 2019, in week 28 at the beginning of July 2020 and, for both years, in week 30 at end of July. After that, a decrease in egg number was observed in September for both years and an increase in egg number was observed later in the season, in both October 2019 and 2020 (week 40). Conversely, the number of collected eggs decreased in October and November and no eggs were observed when average minimal temperatures were below 0 °C. Apart from the local study site in Reichstett, the regional ovitrap survey in the Bas-Rhin administrative unit sampled 5156 eggs in 2019, from week 23 to week 43 (June 3 – October 21, 2019), with an average of 469 ± 387 eggs per two weeks (min: 19 – max: 1078 eggs). In 2020, the Bas-Rhin administrative unit then collected 8610 eggs from week 25 to week 41 (June 15 – October 5, 2020) with an average of 861 ± 813 eggs per two-weeks (min: 221 – max: 2604 eggs) (Table 1). A similar pattern of egg abundance between the study site of Reichstett and at a larger scale in the Bas-Rhin area was observed (Fig. 3), with higher number of eggs at the Bas-Rhin administrative unit in weeks 25, 29 and 43 for 2019, and weeks 29 and 35 for 2020. More precisely, both results of Reichstett and the Bas-Rhin administrative unit showed a bimodal pattern with the first increase occurring from June to August and the second, albeit lower than the previous one, occurring late in October.

**Table 1:** Number of eggs of *Ae. japonicus* collected in Reichstett and in the Bas-Rhin Department. The data from 12 ovitraps for Reichstett and from 158 ovitraps for 2019 to 98 ovitraps for 2020 for the Bas-Rhin Department are summed up per two-weeks.

Reichstett				Bas-Rhin Department			
2019		2020		2019		2020	
Week of collection	Number of eggs	Week of collection	Number of eggs	Week of collection	Number of eggs	Week of collection	Number of eggs
20	16	20	529	21	395		
22	921	22	1592	23	395		
24	3525	24	1243	25	858	25	306
26	3994	26	1489	27	247	27	680
28	2466	28	1698	29	943	29	2604
30	4339	30	3491	31	833	31	1512
32	2338	32	2136	33	1078	33	1073
34	2412	34	1338	35	382	35	1499
36	1112	36	876	37	19	37	487
38	791	38	172	39	34	39	228
40	2448	40	1992	41	152	41	221
42	1336	42	200	43	215	43	0
44	60	44	103				
46	51						
Total	25,809	Total	16,859	Total	5551	Total	8610

The meteorological data analysis was done through PCA. The threshold was set at 75% of explained variance, leading to the retention of the first three components: PC1 (51.4%), PC2 (15.2%) and PC3 (13.7%). In the model retained, 80.3% of variance was thus explained. This analysis showed that egg proportion was highly correlated with the average weekly minimum temperature (*p* = 0.004) and the average weekly maximum temperature (*p* = 0.41) (Data not shown – see Additional File 1, Fig. 1). From weeks 21 to 45, during which almost all eggs were collected, temperatures were on average higher than 7.5 °C, corresponding to the mean of the minimal values of the average minimal temperature (4 °C) and the average maximal temperatures (11 °C), both observed during week 45. During the summer period, an average minimal temperature of 14.2 °C and an average maximal temperature of 27.5 °C were observed, which led to the highest amount of eggs collected. During this period, temperature was 20.8 °C on average. Diapause incidence and week of egg collection were highly correlated (*ρ* = 0.6; *p* < 0.001). Therefore, diapause incidence and average weekly photoperiod were also correlated (*ρ* = −0.65; *p* < 0.001). Another correlation was determined between the average weekly minimum temperature and the diapause incidence (*ρ* = −0.37; *p* = 0.026). Regarding morphology and temperature, we observed that both the volume (*ρ* = −0.65; *p* < 0.001) and width (*ρ* = −0.68; *p* < 0.001) of the eggs were correlated to the average weekly maximum temperature. Rainfall parameters (i.e. the average weekly rainfall and the weekly cumulative rainfall) were not correlated with the number of eggs collected, diapause incidence, nor morphological parameters such as volume or width.

**Figure 4:**
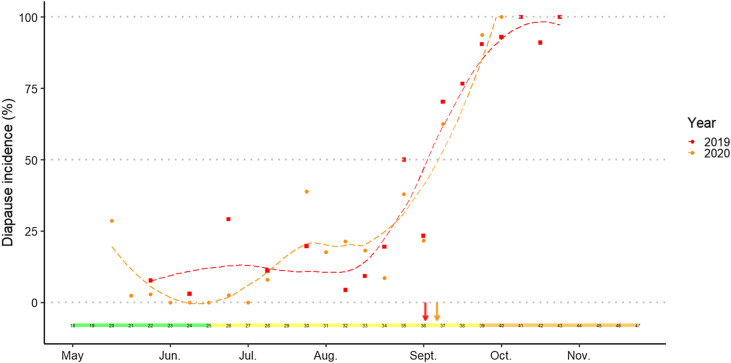
Diapause incidence in *Ae. japonicus* eggs. Mean diapause incidence per week observed from eggs collected in the Reichstett field area. Data for 2019 are shown in empty red triangles while data for 2020 are shown in full orange triangles. Diapause incidence is achieved after hatching tests and bleaching treatment. The curve with dashed lines is the tendency of diapause incidence. A 50% diapause incidence observed in eggs, in dotted grey line, is achieved at week 36 for 2019 (red arrow) and week 37 for 2020 (orange arrow). Corresponding months and seasons are also shown in the coloured horizontal bar (green = spring, yellow = summer, orange = autumn).

Altogether, the partially embryonated and the unfertilised unhatched eggs observed showed a constant mortality rate through the year, with an average of 17.16 ± 19.10% in 2019 and 16.69 ± 29.21% in 2020 on all the eggs tested (Data not shown – see Additional File 1, Fig. 2). An increase in diapause incidence was observed at the beginning of the season in 2019, from the beginning of the egg-laying period to the end of spring (weeks 20–26) with a peak of 13% at week 26 (Fig. 4). Shortly after, a decrease to 10% in diapause incidence on average occurred at week 28. This low rate in diapause incidence was similarly observed in summer from June to August. In early August (week 32), diapause incidence started to increase. A 100% rate of eggs under diapause was eventually reached mid-October, i.e*.* in week 41. Therefore, the 50% cut-off in diapause incidence was reached at week 36, corresponding to a day length of 13 h 07 min at the beginning of September 2019. Similar results were observed for the Bas-Rhin area, meaning that diapause incidence started to increase in mid-July (week 29) and the 50% cut-off of diapause in eggs was reached at week 35 in 2019 (day-length of 13 h 07 min) (Data not shown – see Additional File 2, Fig. 1). In 2020, a similar pattern was observed with an average minimal value of 10% of diapausing eggs at the end of spring, even though some batches of eggs sampled showed 0% diapause incidence. Diapause incidence started to increase during the month of July (week 30) and reached up to 25% in August. Thereafter, diapause incidence increased to 100% at the end of the season, i.e. October. A 50% cut-off in diapause incidence was achieved at a day length of 12 h 49 min at week 37 at the beginning of September 2020.

### Morphological measurements are not consistent throughout the egg-laying period

**Figure 5:**
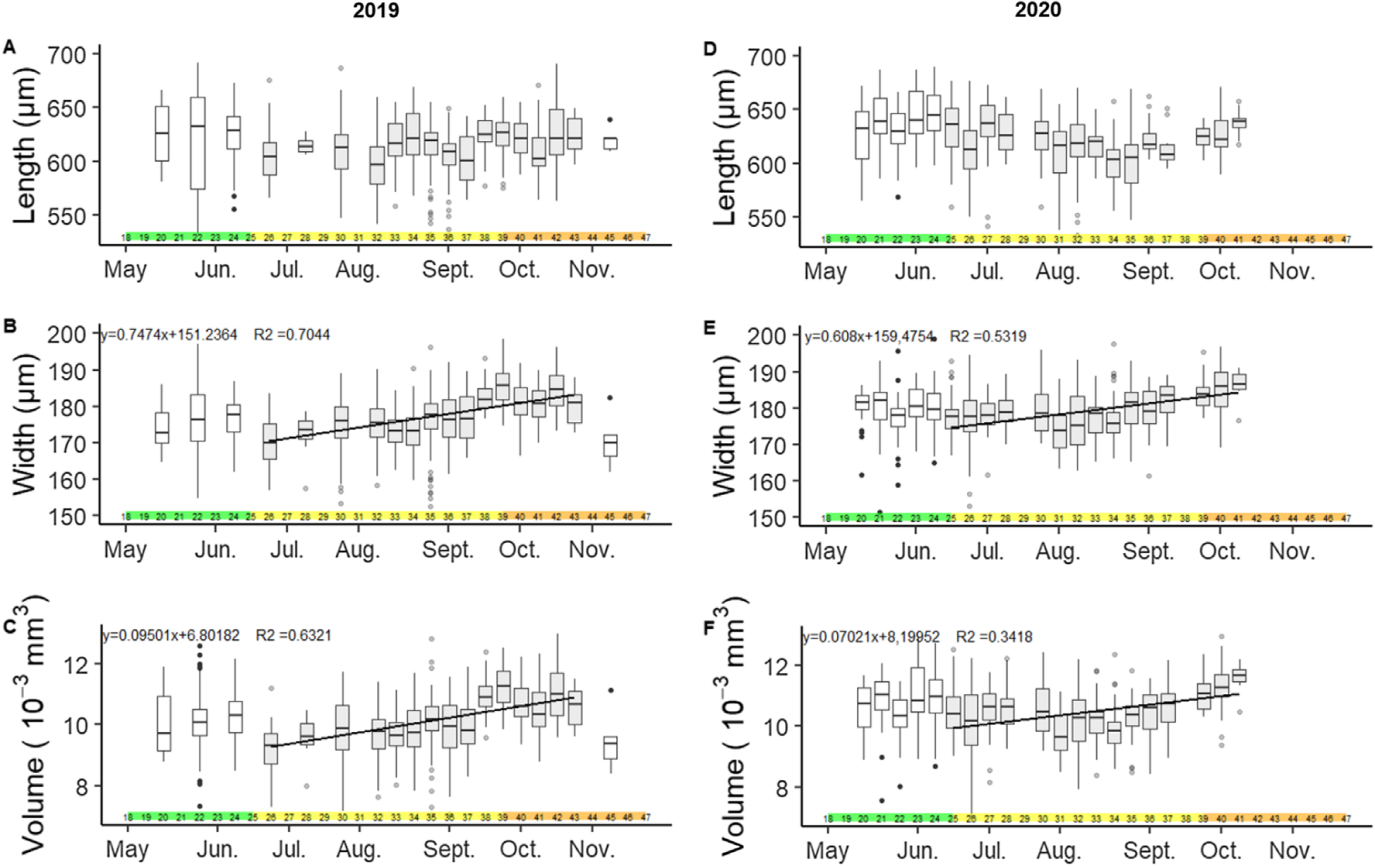
Evolution of morphological parameters of *Ae. japonicus* eggs throughout the year. Figure 5A: length; Figure 5B: width; Figure 5C: volume, data of 2019. Figure 5D: length; Figure 5E: width; Figure 5F: volume, data of 2020. Data come from the Reichstett field area. Eggs are batched according to the calendar week of collection. Corresponding months and seasons in coloured horizontal bars are also shown (green = spring, yellow = summer, orange = autumn). Boxplots of corresponding weeks considered for the linear regression are shown in grey. In Figures 5B and 5C for 2019, linear regressions in solid black lines ignore data point of weeks 20, 22, 24 and 45 as well, which are shown in a white boxplot. For 2020, data for weeks 20–25 are discarded.

Morphological measurements of *Ae. japonicus* eggs showed the following data: median length of 616 μm (IQR: 598–633), median width of 177 μm (IQR: 172–182) and median volume of 10.1 × 10^−3^ mm^3^ (IQR: 9.5–10.8) for 2019 and median length of 624 μm (IQR: 608–642), median width of 179 μm (IQR: 175–183) and median volume of 10.5 × 10^−3^ mm^3^ (IQR: 9.9–11.1) for the year 2020 (Table 2). However, morphological measurements of eggs were not consistent throughout the year. The width and volume of the eggs evolved during the season according to two periods: the first from weeks 20 to 24 and the second later in the season from weeks 26 to 43 (Fig. 5). The morphological evolution through the season was better supported by using the two subsets separately (the two width and volume patterns of the eggs) rather than by mixing all the data (see Additional File 1, Fig. 3). As shown in Figure 5 for 2019, the median values of the eggs were 604 μm in length (IQR: 588–617), 170 μm in width (IQR: 166–175) for a median volume of 9.3 × 10^−3^ mm^3^ (IQR: 8.7–9.7) at the beginning of summer (week 26). At the end of the egg-laying period (week 43), median length increased to 621 μm (IQR: 612–640), median width to 181 μm (IQR: 176–183) for a median volume of 10.7 × 10^−3^ mm^3^ (IQR: 9.9–11.1). As a result, the volume of the eggs increased by 14.6% between week 26 and week 43 in 2019. Similarly, volume increased by 15.05% between weeks 26 and 41 in 2020.

**Table 2:** Details of morphological metrics measured on *Ae. japonicus* eggs. Data originate from the Reichstett field area. Length and width of eggs are measured three times per egg.

	Length (μm)	Width (μm)	Volume (×10^−3^ mm^3^)
2019 (*n* = 1241)			
Minimum	489	151	6.6
Maximum	727	203	13.8
Mean	615	177	10.1
Standard-deviation	29	8	1.1
First quartile	598	172	9.5
Median	616	177	10.1
Third quartile	633	182	10.8
2020 (*n* = 777)			
Minimum	533	145	6.5
Maximum	710	206	14.1
Mean	625	179	10.5
Standard-deviation	27	7	1.0
First quartile	608	175	9.9
Median	624	179	10.5
Third quartile	642	183	11.1

Due to measurement and calculation methods, the length and width of eggs were weakly correlated (*ρ* = 0.10; *p* = 7.21 × 10^−4^). Volume was highly correlated to length and width of eggs, with *ρ* = 0.56; *p* < 2.2 × 10^−16^ and *ρ* = 0.88; *p* < 2.2 × 10^−16^, respectively. Despite a high variability in length parameters of eggs (range: 488.71–727.20 μm – see Table 2), no significant correlation between length of eggs and week of collection was observed (*p* = 0.66). On the contrary, both the width and volume of the eggs increased according to the week of collection (width: *ρ* = 0.51; *p* = 0.03 and linear regression of width (μm) = 0.7474 × week + 151.24; *R*^2^ = 0.71; *p* = 5.58 × 10^−5^ and volume: *ρ* = 0.47; *p* = 4.39 × 10^−2^ and linear regression of volume (10^−3^ mm^3^) = 0.095 × week + 6.80; *R*^2^ = 0.63; *p* = 2.40 × 10^−4^).

Outside of the study area, at a larger scale in the Bas-Rhin region, the eggs collected in the late season were also wider and larger in volume than the eggs sampled earlier (Data not shown – see Additional File 2, Fig. 2).

### Width measurement as a predictor to evaluate diapause incidence in *Ae. japonicus* eggs

**Figure 6:**
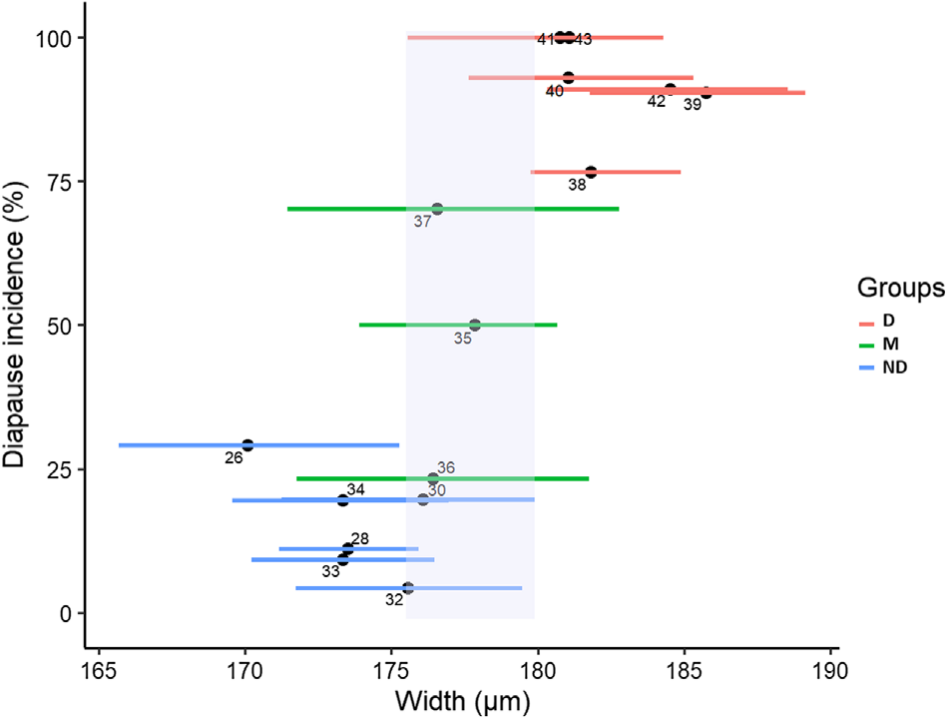
Association between the width of the eggs and diapause incidence. Data come from 2019 and the Reichstett field area. Eggs are batched according to the calendar week of collection. For each week, average diapause incidence is used. Width of eggs is shown as dots for median values and lines as interquartile ranges. Three groups are defined depending on their week of collection: group ND from weeks 26 to 34 (in blue), group M from 35 to 37 (in green) and group D from 38 to 43 (in red). An overlap in width between group ND and D is highlighted in pale lavender.

The 50% diapause incidence cut-off was observed on week 36 in 2019 and week 37 in 2020. Thus, group M gathers eggs sampled between weeks 35–37 for 2019 and 36–38 for 2020. The diapause incidence of group ND, which was composed of eggs collected between weeks 26–34 for 2019 and weeks 26–36 for 2020, was below 30%. Width of eggs ranged from 145 to 198 μm and was 176 ± 7 μm on average. Eggs sampled from weeks 38 to 43 gathered in group D had a diapause incidence higher than 75% in 2019. In 2020, group D included eggs collected from weeks 40 to 43. Mean width of eggs in group D was 183 ± 6 μm within the range of 162–198 μm. As a result, eggs in group D were 4.26% wider (Wilcoxon test, *W* = 237,016, *p* < 2.2 × 10^−16^) than those of group ND. The volume of the eggs was 10.17% higher in group D than in group ND (Wilcoxon test, *W* = 233,679, *p* < 2.2 × 10^−16^) (see Additional File 1, Table 2). Nevertheless, an overlap between 162 and 198 μm was observed in egg width (Fig. 6) and included eggs from groups ND, D and M each corresponding to a range from 7 to 100% in diapause incidence.

**Figure 7:**
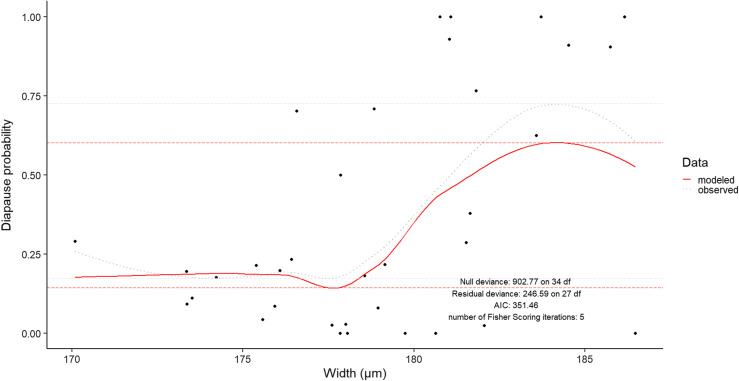
Field data and predicted model of diapause probability. Data come from the Reichstett field area. Mean per week is shown in black dots. Grey dotted line is the associated curve trend. A logistic model of regression, considering week of collection, year and width of eggs is shown with a solid red line. Diapause incidence is in both cases higher when width measurements increase. Minimum and maximum values of observed diapause probability and predicted diapause probability are highlighted in horizontal long dashed lines, in grey and red, respectively. Model estimators are directly written on the plot.

The proposed model was mainly explained by week of collection (*p* = 1.27 × 10^−10^), width of eggs (*p* = 6.61 × 10^−12^), year (*p* = 9.77 × 10^−11^), and the interaction between these factors (Fig. 7). Neither a null probability nor a rate of 100% in diapause incidence was expressed by the model. Categorisation into two groups was therefore not possible. In addition, a range of 162–197 μm in the width of eggs and from 17 to 72% in diapause incidence was observed in field data. The model predicted a diapause incidence of 14–60% for the same width range.

## Discussion

Dormancy is a key parameter for the overwintering of Culicid species in temperate regions. However, even though highly suspected, the diapause incidence of *Ae. japonicus* in Europe was unknown. This study shows that successive stimuli like water immersion and temperatures did not lead to the hatching of eggs, while fully developed embryos were observed in these eggs after a bleaching treatment. This study thus confirms effective diapause for *Ae. japonicus*. In the case of quiescence, eggs would have hatched under such stimuli. Interestingly, a diapause incidence of around 10% was observed throughout the whole egg-laying season both years meaning that female *Ae. japonicus* always lay part of their eggs in a diapause state, regardless of the meteorological and photoperiodic conditions. This study thus found that diapause, a phenomenon used to survive harsh winter conditions in our region, in fact already started at the beginning of the oviposition season. Since cold temperatures can inhibit larvae development [35, 42], we can hypothesise that the diapause status of these eggs laid in the early season can be a way to survive hazardous weather conditions, given that some freezing events can still occur during the month of May in the northeast of France. Even though larvae can pursue their development during winter when temperatures are favourable, diapausing eggs appears to be a strategy to guarantee the survival of the species in fluctuating weather conditions in temperate regions. This study suggests that this characteristic is important for the survival of this species since the diapause phenomenon occurs throughout the year.

We observed an increase in diapause during mid-summer, three months before the end of the *Ae. japonicus* oviposition season. While a 50% diapause incidence in eggs was detected in September both years, mid-October was determined as the period in which all eggs were under diapause. A one-week variation between the 50% cut-off in 2019 and 2020 was nevertheless observed in the studied field population. A similar study on *Ae. albopictus* showed no difference in diapause incidence nor in the volume of eggs between two consecutive years in the field [26]. This variation reinforces the idea that the synergy between photoperiod and temperatures together trigger and control the diapause phenomenon in this species. Regarding the influence of photoperiod on diapause, we observed a 50% diapause incidence of eggs at week 36 in 2019 and at week 37 in 2020, corresponding to a day length of 13 h 07 min and 12 h 49 min, respectively. Similarly, in the south of France in 2010 and 2011, week 36 with a day length of 12 h 41 min was mentioned as the critical period that induces a 50% diapause in eggs of *Ae. albopictus* [26]. Female adults of *Ae. japonicus*, and pupa to a lesser extent, are receptive to photoperiod to induce diapause in eggs [7]. The maternal day-length when 50% of the eggs laid are under diapause, is called the critical photoperiod (CPP). In *Ae. albopictus,* another invasive species also found in the northeast of France, the CPP determined in laboratory conditions was 13 h 30 min [26]. Our results in the field suggest that the CPP may vary between 13 h 31 min and 13 h 14 min for 2019 and 2020, respectively, but could increase to 13 h 57 min or 13 h 38 min if a two-week delay is taken into account as suggested by Lacour et al. [26]. As these authors suggest, the delay between the CPP observed in females and the 50% cut-off in eggs in the field may be due to environmental factors and physiological delay [26]. Eggs from the previous egg-laying season (i.e. 2018 and then 2019) seem to hatch at the end of February or the beginning of March. Consequently, we estimate the duration of diapause to be three months at least. Moreover, we found a correlation between diapause incidence and both width and volume in eggs. We also found that diapause incidence varied throughout the year and was even found at low levels at the beginning of the season, suggesting that photoperiod is not the only factor to induce diapause. Temperatures might also play a role and induce diapause [37]. On the whole, the results in this study suggest that within the same field population, both diapause incidence and the volume of the eggs increase while temperatures decrease.

With regard to seasonality of *Ae. japonicus* in our studied area, we showed that the period of the egg laying activity ran from the end of May to the end of October, and the beginning of November in the second year. This general pattern of adult activity observed in East Asian Bush mosquitoes in the colonised area is quite consistent with other studies in native areas [18, 48] as well as in colonised areas [15]. In Belgium [54], authors observed young larvae prior to the egg laying period, suggesting that *Ae. japonicus* eggs are thus likely to hatch at cooler temperatures and earlier than other invasive species like *Ae. albopictus*. As for the meteorological parameters linked to *Ae. japonicus* activity in this study, seasonal temperatures turned out to be the main parameter influencing egg abundance. A previous study showed that cooler temperatures increased *Ae. japonicus* presence in larval habitats [4]. Our results suggest that eggs are laid when temperatures are above 7.5 °C. A similar temperature threshold was evidenced by thermal experiments in laboratory conditions in which 7 °C was determined as the critical minimal temperature allowing larvae development [35]. In Japan, a field study including an altitudinal gradient found that fourth instar larvae were absent in temperatures below 10 °C [8]. This study proposed a model which predicted that a minimal temperature of 12.41 °C was necessary to reach fourth instar stage development. We also observed more eggs in the summer months (July and August) and later in October in our area of study. This corroborates the findings of Murrell et al. [31], who found that *Ae. japonicus* in North America laid more eggs in July than in September. A possible explanation for the decrease in the number of eggs laid may be the inhibition of larval development when temperatures rise above 34 °C and up to 40 °C [42]. In the case of our study, such an observation was made in August and September 2019 when heat waves with temperatures frequently above 30 °C preceded a decrease in egg abundance.

On average, the eggs measured in this study were 29 μm longer and 5 μm wider than those of a previous study [16]. This difference may reflect the origin of the eggs. In our study, these were collected in the field, whereas the egg measurements in the previous study came from a colony. More precisely, this *Ae. japonicus* colony was derived from specimens collected in the field (NJ, USA) and reared in an insectarium for 8–9 years [16]. A study on another species, namely *Ae. aegypti,* showed size variations between eggs from the field and those from different laboratory colonies [14], suggesting that the origin of samples can have an incidence on egg morphology. Our study, which only focuses on eggs of *Ae. japonicus,* showed that width was the main factor of the volume variability of the eggs. Similar relationships between width and volume variability were demonstrated for *Ae. albopictus* eggs [25]. Egg morphometric analyses frequently showed intraspecific variation between mosquitoes collected in different places [14, 28]. For *Ae. aegypti,* it has been shown that an increase in the sizes of either the female body or the blood meal produce larger eggs [46]. Furthermore, the volume of the eggs was correlated with survival capacity suggesting that larger eggs survive for longer periods of time [45]. Since eggs are bigger in autumn than in spring, which corresponds to the period in which they prepare for the overwintering period, local environmental conditions are likely to impact egg size as well as diapause status.

Since a relationship between morphometrics and the diapause status was evidenced for *Ae. albopictus* [25], we hypothesised that this diapause phenomenon in *Ae. japonicus* would also induce a phenotype in eggs that could be detected by morphological measurements. The increase in egg size was shown to be linked to a gain in the quantity of lipids for *Ae. albopictus* [25]. Therefore diapause, as a strategy to survive harsh conditions, could lead to a seasonal phenotype in adults. While we showed a positive correlation between diapause incidence and eggs size, the width and volume measurements cannot ascertain the diapause status of eggs. More precisely, our model neither revealed a rate of 0% nor 100% in diapause incidence but showed a minimum of approximately 10% of eggs under diapause even at the very beginning of the season. As a consequence, an overlap in morphological measurements exists between diapaused and non-diapaused eggs suggesting that, despite being positively correlated, factors other than diapause affect the volume of eggs. For instance, factors like physiological status, size of females and the amount of blood meals can alter the volume of eggs [46].

On the whole, the seasonality in diapause incidence for the Asian bush mosquito was described here at the intra-population level. Studying the influence of temperature on the oviposition behaviour of *Ae. japonicus* would greatly help to increase knowledge of its seasonality and could eventually lead to the development of a predictive model of invasion. In addition to field studies, laboratory experiments on *Ae. japonicus* colonies would be helpful to understand parameters such as egg morphology under various temperatures and day-length effects. The use of climatic chambers to investigate diapause would help to define CPP more precisely in *Ae. japonicus.* Similar experiments can be used to investigate factors of preparation, duration and termination of diapause under laboratory conditions. Moreover*,* some mechanisms observed in *Ae. albopictus* such as lipid storage in diapause eggs or genetic determination of diapause [11] have yet to be investigated for *Ae. japonicus.*

In this study, we demonstrated effective diapause of the East Asian Bush mosquito in the northeast of France and described the morphology and the diapause status of eggs in a field population. More particularly, this study showed that diapause eggs were laid during the entire oviposition season, even at the end of spring, provided that a mean value of 10% of diapause incidence was observed. While the oviposition period of *Ae. japonicus* ran from May to November, a 50% diapause incidence was determined between late August and early September. More precisely, diapause was found to increase when the photoperiod decreased and the subsequent maternal CPP was determined at 13 h 23 min. Other factors such as cold temperatures may induce an increase of diapause incidence and further laboratory experiments would be required. Regarding morphology, in this study we observed an increase in egg volume through the season. While a positive correlation was shown between both volume and width of eggs and the diapause status, the measurement of egg morphology cannot be used to predict it. Diapause and seasonality are one of many strategies involved in the successful invasion of *Ae. japonicus.* This study provides insights on their characterisation and improves our current knowledge of the invasive traits of the East Asian bush mosquito. Our findings could help in future predictive modelling for invasive species such as these.

## Abbreviations

AIC: Akaike information criterion
CPP: Critical photoperiod
*df*: Degree of freedom
IQR: Interquartile range
*p*: *p*-value

## Supplementary Materials

Supplementary material is available at https://www.parasite-journal.org/10.1051/parasite/2021045/olm *Additional File 1 – Table 1*. Sample size for morphological measurement, hatching success rate, mortality rate and diapause incidence. Data originate from the Reichstett field area.
*Additional File 1 – Figure 1*. Environmental parameters PCA. The first three components were retained: PC1 (51.4%), PC2 (15.2%) and PC3 (13.7%) and thus, 80.3% of variance was explained. Variables are projected in a plan formed by PC1 and PC2. All variables are projected in light grey, some are highlighted for better visualisation. From left to right and up to down: A, week number and year are in black and diapause incidence is in purple; B, morphological parameters, i.e. median width and median volume are in green; C, maximal temperature parameters are in red; D, minimal temperature parameters are in blue; E, light parameters, photoperiod is showed in orange and sunshine parameters are in yellow; F, rainfall parameters are in dark blue.
*Additional File 1 – Figure 2*. Mortality rate of *Aedes japonicus* eggs. Data originate from the Reichstett field area. Percentage of unsustainable eggs for each week are showed as black dots. Mean with standard-deviation is shown by a solid black line surrounded by grey. The mortality rate was on average 17.16% in 2019 and 16.69% in 2020. The corresponding months and seasons are also shown on the coloured horizontal bar (green = spring, yellow = summer, orange = autumn).
*Additional File 1 – Figure 3*. Example of two linear regressions between the width of eggs and the week of collection. Only the data for the 2019 season from the Reichstett field area are shown. Data for week 45 are discarded due to an insufficient sample size (*n* = 5). In panel A, all data (shown in red) are gathered in one dataset for linear regression. Adjusted *R*^2^ is 17.69%. In panel B, data are subdivided in two datasets, from weeks 20 to 24 (in blue) and weeks 26 to 43 (in grey). Linear regressions are better fitted with these two data subsets (adjusted *R*^2^ 84.63% and 70.44%). The corresponding months and seasons are also shown on the coloured horizontal bar (green = spring, yellow = summer, orange = autumn).
*Additional File 1 – Table 2*. Morphological parameters on two groups of eggs. Data originate from the Reichstett field area. Group ND is chosen as the baseline for comparisons with group D. An increase of each morphological parameter is observed. Wilcoxon Sum rank tests were performed. Two stars represent a significant increase between groups ND and D.
*Additional File 2 – Figure 1*. Diapause incidence in *Ae. japonicus* eggs. Red dots represent the mean diapause incidence per week observed in eggs collected in the Bas-Rhin region in 2019. Diapause incidence was achieved after performing hatching tests and a bleaching treatment. The curve with the dashed red lines is the tendency of diapause incidence. The 50% diapause incidence observed in eggs at week 35 in 2019 is shown as a dotted grey line. Data for 2020 are not shown due to insufficient data. Corresponding months and seasons are also shown in the coloured horizontal bar (green = spring, yellow = summer, orange = autumn).
*Additional File 2 – Figure 2*. Changes in morphological parameters of *Ae. japonicus* eggs throughout the year. Figure 2A Length; Figure 2B Width; Figure 2C Volume, data of 2019. Figure 2D Length; Figure 2E Width; Figure 2F Volume, data for 2020. Data originate from the Bas-Rhin region. Eggs are batched according to the calendar week of collection. Corresponding months and seasons are also shown in the coloured horizontal bars (green = spring, yellow = summer, orange = autumn). Data for week 37 in 2019 are discarded.
*Additional File 2 – Table 1*. Details of morphological metrics measured on *Ae. japonicus* eggs. Data originate from the Bas-Rhin region. Length and width are measured 3 times per egg.
*Additional File 2 – Table 2*. Sample size for morphological measurements, hatching success rate, mortality rate and diapause incidence. Data come from the Bas-Rhin region.

## Competing interests

The authors declare no competing interests.
