# Supplementary material for: Diapause characterisation and seasonality of Aedes japonicus japonicus (Diptera, Culicidae) in the northeast of France
Source: Parasite. 2021 May 26;28:45. doi: 10.1051/parasite/2021045 (PMC8152802; doi:10.1051/parasite/2021045)
Supplement: Supplementary file 1 — Additional File 1 – Table 1. Sample size for morphological measurement, hatching success rate, mortality rate and diapause incidence. Data originate from the Reichstett field area. Additional File 1 – Figure 1. Environmental parameters PCA. The first three components were retained: PC1 (51.4%), PC2 (15.2%) and PC3 (13.7%) and thus, 80.3% of variance was explained. Variables are projected in a plan formed by PC1 and PC2. All variables are projected in light grey, some are highlighted for better visualisation. From left to right and up to down: A, week number and year are in black and diapause incidence is in purple; B, morphological parameters, i.e. median width and median volume are in green; C, maximal temperature parameters are in red; D, minimal temperature parameters are in blue; E, light parameters, photoperiod is showed in orange and sunshine parameters are in yellow; F, rainfall parameters are in dark blue. Additional File 1 – Figure 2. Mortality rate of Aedes japonicus eggs. Data originate from the Reichstett field area. Percentage of unsustainable eggs for each week are showed as black dots. Mean with standard-deviation is shown by a solid black line surrounded by grey. The mortality rate was on average 17.16% in 2019 and 16.69% in 2020. The corresponding months and seasons are also shown on the coloured horizontal bar (green = spring, yellow = summer, orange = autumn). Additional File 1 – Figure 3. Example of two linear regressions between the width of eggs and the week of collection. Only the data for the 2019 season from the Reichstett field area are shown. Data for week 45 are discarded due to an insufficient sample size (n = 5). In panel A, all data (shown in red) are gathered in one dataset for linear regression. Adjusted R2 is 17.69%. In panel B, data are subdivided in two datasets, from weeks 20 to 24 (in blue) and weeks 26 to 43 (in grey). Linear regressions are better fitted with these two data subsets (adjusted R2 84.63% and 70.44%). The correspo [file parasite-28-45-s1.zip › parasite210011-1-olm/22032021_diapause_japo_additional_file_1.docx]

**Additional file 1 – Table 1. Sample size for morphological measurement, hatching success rate, mortality rate and diapause incidence.** Data originate from the Reichstett field area.

| 2019 | Number of eggs | | | |
| --- | --- | --- | --- | --- |
|  | Collected | Measured | Tested after hatching | Bleached |
| Week |  |  |  |  |
| 20 | 16 | 14 | 14 | 0 |
| 22 | 921 | 151 | 227 | 46 |
| 24 | 3,525 | 86 | 143 | 13 |
| 26 | 3,994 | 37 | 66 | 27 |
| 28 | 2,466 | 9 | 12 | 4 |
| 30 | 4,339 | 84 | 89 | 28 |
| 32 | 2,338 | 104 | 123 | 13 |
| 33 | 1,020 | 86 | 80 | 11 |
| 34 | 1,392 | 136 | 141 | 42 |
| 35 | 414 | 105 | 114 | 65 |
| 36 | 698 | 104 | 86 | 40 |
| 37 | 437 | 38 | 40 | 29 |
| 38 | 354 | 45 | 100 | 82 |
| 39 | 962 | 49 | 69 | 63 |
| 40 | 1,486 | 79 | 77 | 73 |
| 41 | 460 | 32 | 31 | 31 |
| 42 | 876 | 69 | 115 | 108 |
| 43 | 60 | 12 | 15 | 15 |
| 44 | 0 | 0 | 0 | 0 |
| 45 | 51 | 5 | 5 | 5 |
| 2020 | Number of eggs | | | |
|  | Collected | Measured | Tested after hatching | Bleached |
| week |  |  |  |  |
| 20 | 529 | 19 | 23 | 13 |
| 21 | 617 | 31 | 48 | 7 |
| 22 | 975 | 50 | 39 | 5 |
| 23 | 537 | 39 | 32 | 0 |
| 24 | 706 | 70 | 38 | 2 |
| 25 | 356 | 51 | 38 | 0 |
| 26 | 1,133 | 63 | 40 | 2 |
| 27 | 1,309 | 46 | 37 | 0 |
| 28 | 389 | 31 | 27 | 4 |
| 30 | 3,491 | 45 | 61 | 50 |
| 31 | 862 | 39 | 38 | 10 |
| 32 | 1,274 | 56 | 46 | 13 |
| 33 | 670 | 28 | 34 | 7 |
| 34 | 668 | 66 | 51 | 8 |
| 35 | 511 | 29 | 29 | 11 |
| 36 | 365 | 25 | 24 | 6 |
| 37 | 172 | 12 | 13 | 10 |
| 39 | 1,323 | 19 | 20 | 19 |
| 40 | 669 | 28 | 30 | 30 |
| 41 | 76 | 10 | 10 | 0 |
| 42 | 124 | 0 | 0 | 0 |
| 43 | 0 | 0 | 0 | 0 |
| 44 | 103 | 0 | 0 | 0 |


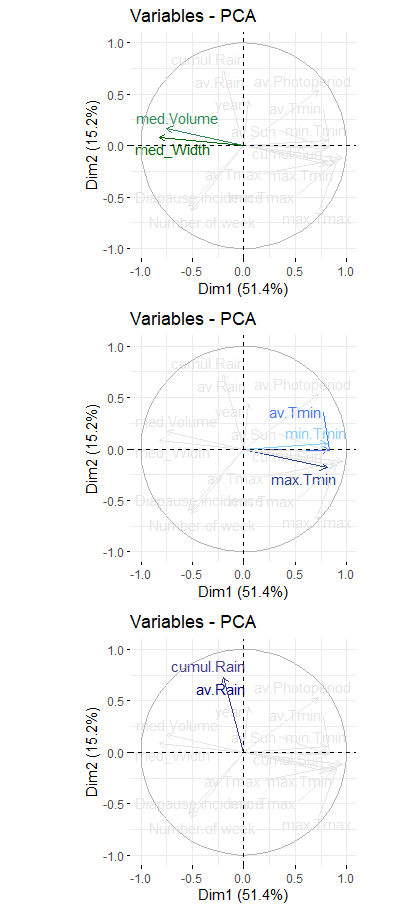


**B**

**D**

**F**


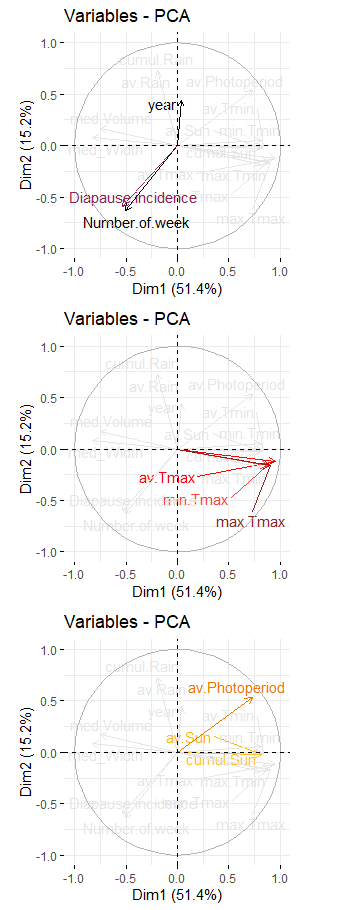


**A**

**C**

**E**

**Additional file 1 – Figure 1. Environmental parameters PCA**. The first three components were retained: PC1 (51.4%), PC2 (15.2%) and PC3 (13.7%) and thus, 80.3% of variance was explained. Variables are projected in a plan formed by PC1 and PC2. All variables are projected in light grey, some are highlighted for better visualisation. From left to right and up to down: A, week number and year are in black and diapause incidence is in purple; B, morphological parameters, i.e. median width and median volume are in green; C, maximal temperature parameters are in red; D, minimal temperature parameters are in blue; E, light parameters, photoperiod is showed in orange and sunshine parameters are in yellow; F, rainfall parameters are in dark blue.


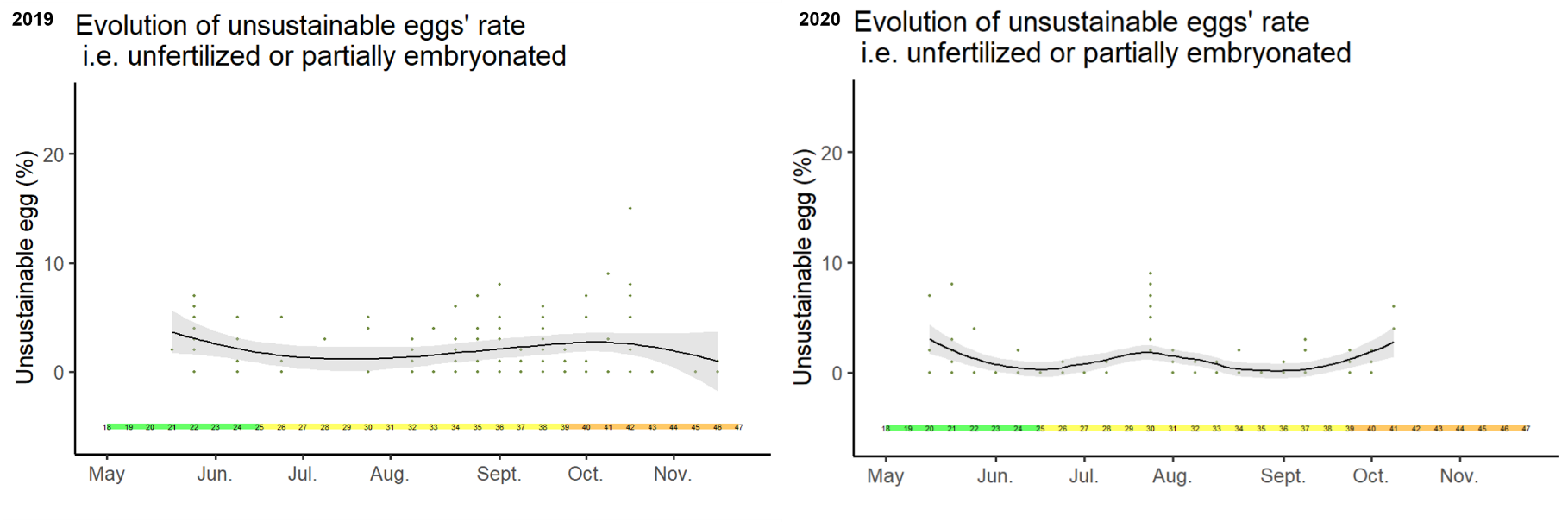


**Additional file 1 – Figure 2. Mortality rate of *Aedes japonicus* eggs.** Data originate from the Reichstett field area. Percentage of unsustainable eggs for each week are showed as black dots. Mean with standard-deviation is shown by a solid black line surrounded by grey. The mortality rate was on average 17.16% in 2019 and 16.69% in 2020. The corresponding months and seasons are also shown on the coloured horizontal bar (green=spring, yellow=summer, orange=autumn).


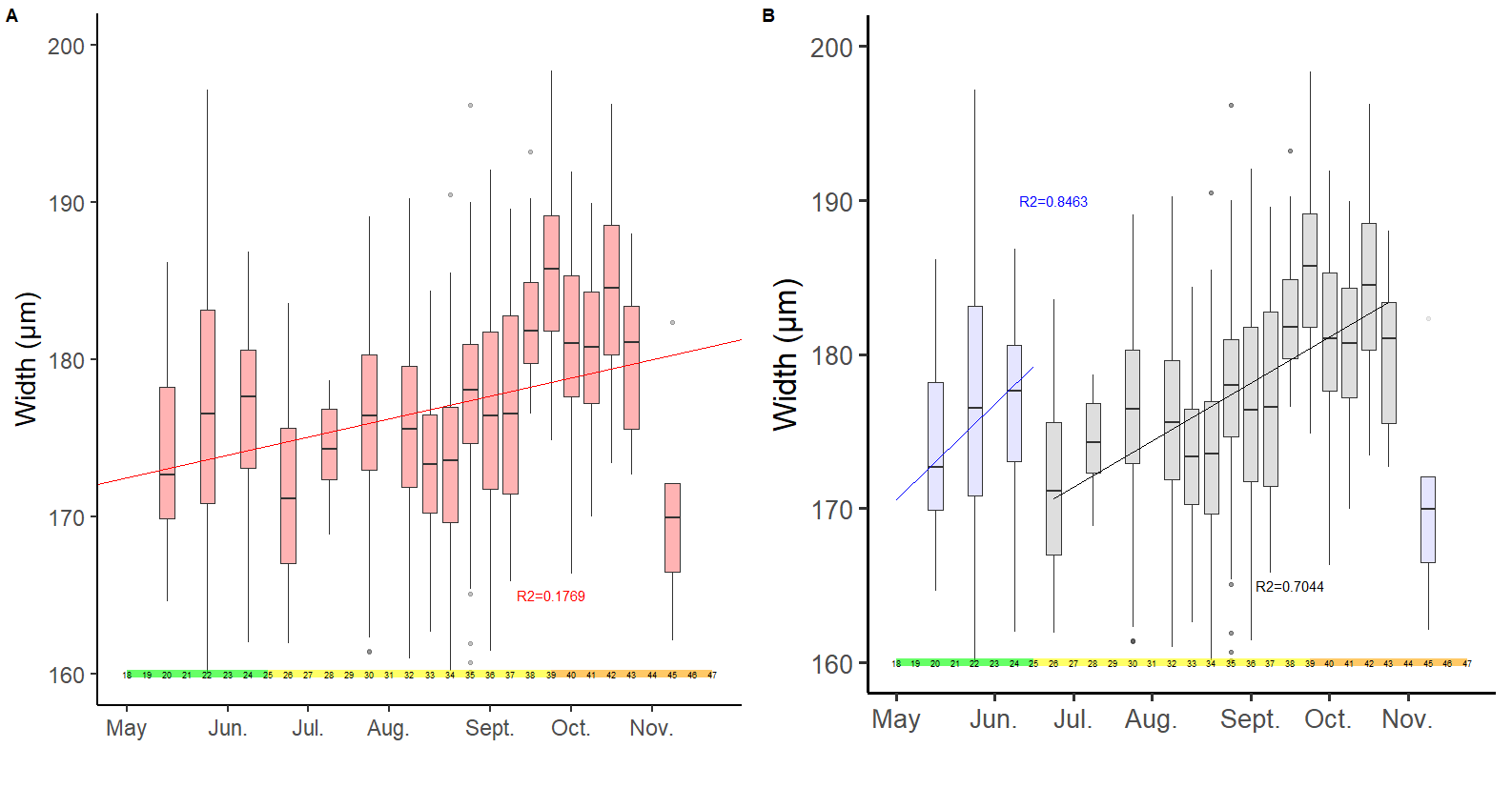


**Additional file 1 – Figure 3. Example of two linear regressions between the width of eggs and the week of collection**. Only the data for the 2019 season from the Reichstett field area are shown. Data for week 45 are discarded due to an insufficient sample size (n=5). In panel A, all data (shown in red) are gathered in one dataset for linear regression. Adjusted R^2^ is 17.69%. In panel B, data are subdivided in two datasets, from weeks 20 to 24 (in blue) and weeks 26 to 43 (in grey). Linear regressions are better fitted with these two data subsets (adjusted R^2^ 84.63% and 70.44%). The corresponding months and seasons are also shown on the coloured horizontal bar (green=spring, yellow=summer, orange=autumn).

**Additional file 1 – Table 2. Morphological parameters on two groups of eggs.** Data originate from the Reichstett field area. Group ND is chosen as the baseline for comparisons with group D. An increase of each morphological parameter is observed. Wilcoxon Sum rank tests were performed. Two stars represent a significant increase between groups ND and D.

| 2019 |  |  |  |  |  |
| --- | --- | --- | --- | --- | --- |
| Batch of eggs | Group ND |  | Group D |  | Increase (%) between groups ND and D |
| Week of collection | [26] only | [26 – 34] | [38 – 43] | [43] only |  |
| n=  Length (µm) | 37  604 ± 24 | 455  612 ± 27 | 291  623 ± 22 | 17  624 ± 18 | 1.8 %** |
| Width (µm) | 170 ± 7 | 174 ± 6 | 183 ± 5 | 176 ± 5 | 5.3 % ** |
| Volume (×10^-3^ mm^3^) | 9.2 ± 0.8 | 9.7 ± 0.9 | 10.9 ± 0.8 | 10.6 ± 0.7 | 12.8 %** |
| 2020 |  |  |  |  |  |
| Batch of eggs | Group ND |  | Group D |  | Increase (%) between groups ND and D |
| Week of collection | [26] only | [26 – 35] | [40 – 41] | [41] only |  |
| n=  Length (µm) | 64  615 ± 28 | 409  618 ± 26 | 40  627 ± 17 | 11  630 ± 17 | 1.5 % ** |
| Width (µm) | 176 ± 9 | 177 ± 7 | 185 ± 7 | 186 ± 6 | 4.6 % ** |
| Volume (×10^-3^ mm^3^) | 10.1 ± 1.3 | 10.2 ± 1.0 | 11.3 ± 0.8 | 11.4 ± 0.7 | 10.9 % ** |
