# Supplementary material for: Diapause characterisation and seasonality of Aedes japonicus japonicus (Diptera, Culicidae) in the northeast of France
Source: Parasite. 2021 May 26;28:45. doi: 10.1051/parasite/2021045 (PMC8152802; doi:10.1051/parasite/2021045)
Supplement: Supplementary file 1 — Additional File 1 – Table 1. Sample size for morphological measurement, hatching success rate, mortality rate and diapause incidence. Data originate from the Reichstett field area. Additional File 1 – Figure 1. Environmental parameters PCA. The first three components were retained: PC1 (51.4%), PC2 (15.2%) and PC3 (13.7%) and thus, 80.3% of variance was explained. Variables are projected in a plan formed by PC1 and PC2. All variables are projected in light grey, some are highlighted for better visualisation. From left to right and up to down: A, week number and year are in black and diapause incidence is in purple; B, morphological parameters, i.e. median width and median volume are in green; C, maximal temperature parameters are in red; D, minimal temperature parameters are in blue; E, light parameters, photoperiod is showed in orange and sunshine parameters are in yellow; F, rainfall parameters are in dark blue. Additional File 1 – Figure 2. Mortality rate of Aedes japonicus eggs. Data originate from the Reichstett field area. Percentage of unsustainable eggs for each week are showed as black dots. Mean with standard-deviation is shown by a solid black line surrounded by grey. The mortality rate was on average 17.16% in 2019 and 16.69% in 2020. The corresponding months and seasons are also shown on the coloured horizontal bar (green = spring, yellow = summer, orange = autumn). Additional File 1 – Figure 3. Example of two linear regressions between the width of eggs and the week of collection. Only the data for the 2019 season from the Reichstett field area are shown. Data for week 45 are discarded due to an insufficient sample size (n = 5). In panel A, all data (shown in red) are gathered in one dataset for linear regression. Adjusted R2 is 17.69%. In panel B, data are subdivided in two datasets, from weeks 20 to 24 (in blue) and weeks 26 to 43 (in grey). Linear regressions are better fitted with these two data subsets (adjusted R2 84.63% and 70.44%). The correspo [file parasite-28-45-s1.zip › parasite210011-1-olm/22032021_diapause_japo_additional_file_2.docx]

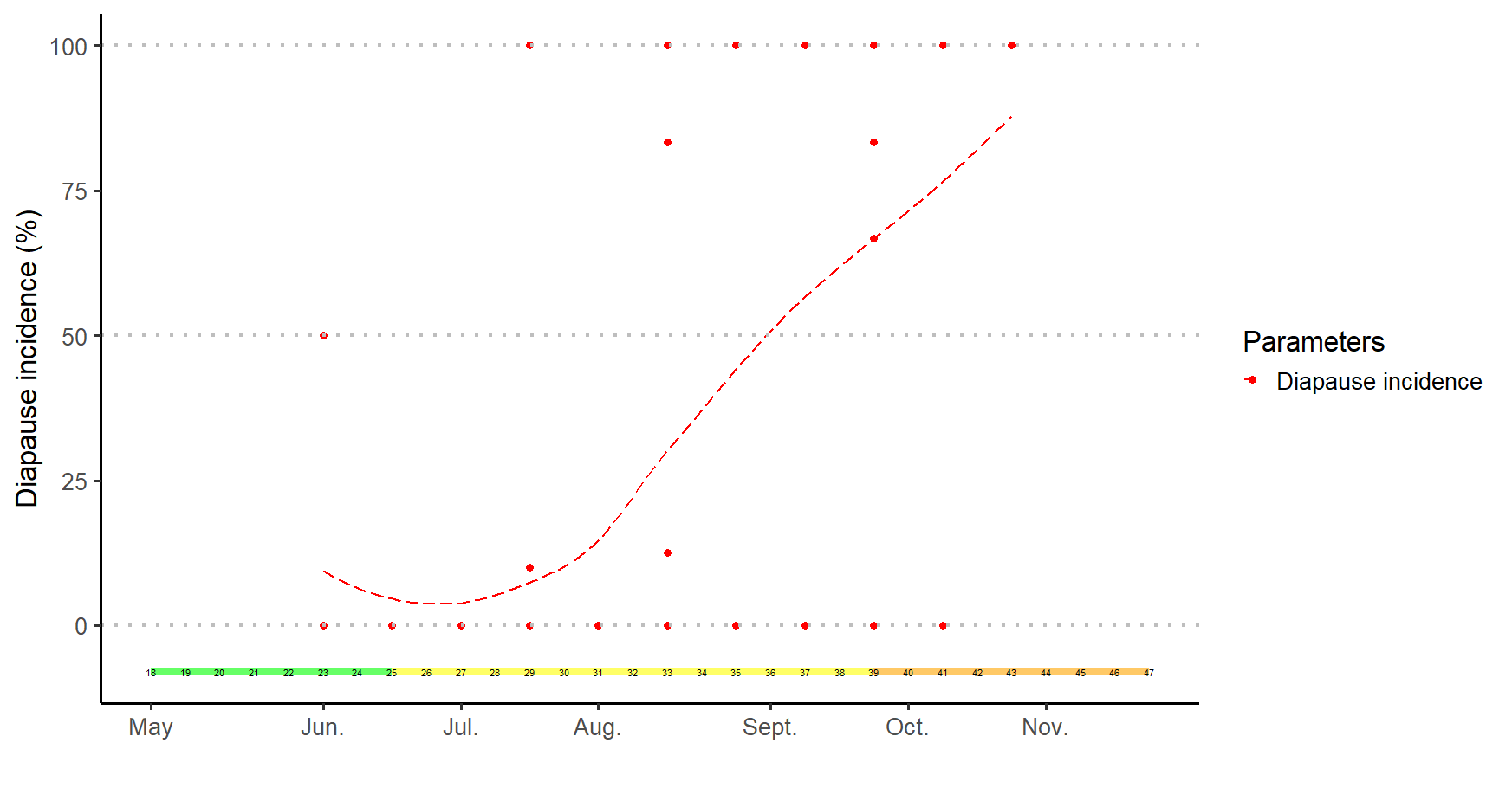


**Additional file 2. Figure 1. Diapause incidence in *Ae. japonicus* eggs.** Red dots represent the mean diapause incidence per week observed in eggs collected in the Bas-Rhin region in 2019. Diapause incidence was achieved after performing hatching tests and a bleaching treatment. The curve with the dashed red lines is the tendency of diapause incidence. The 50% diapause incidence observed in eggs at week 35 in 2019 is shown as a dotted grey line. Data for 2020 are not shown due to insufficient data. Corresponding months and seasons are also shown in the coloured horizontal bar (green=spring, yellow=summer, orange=autumn).


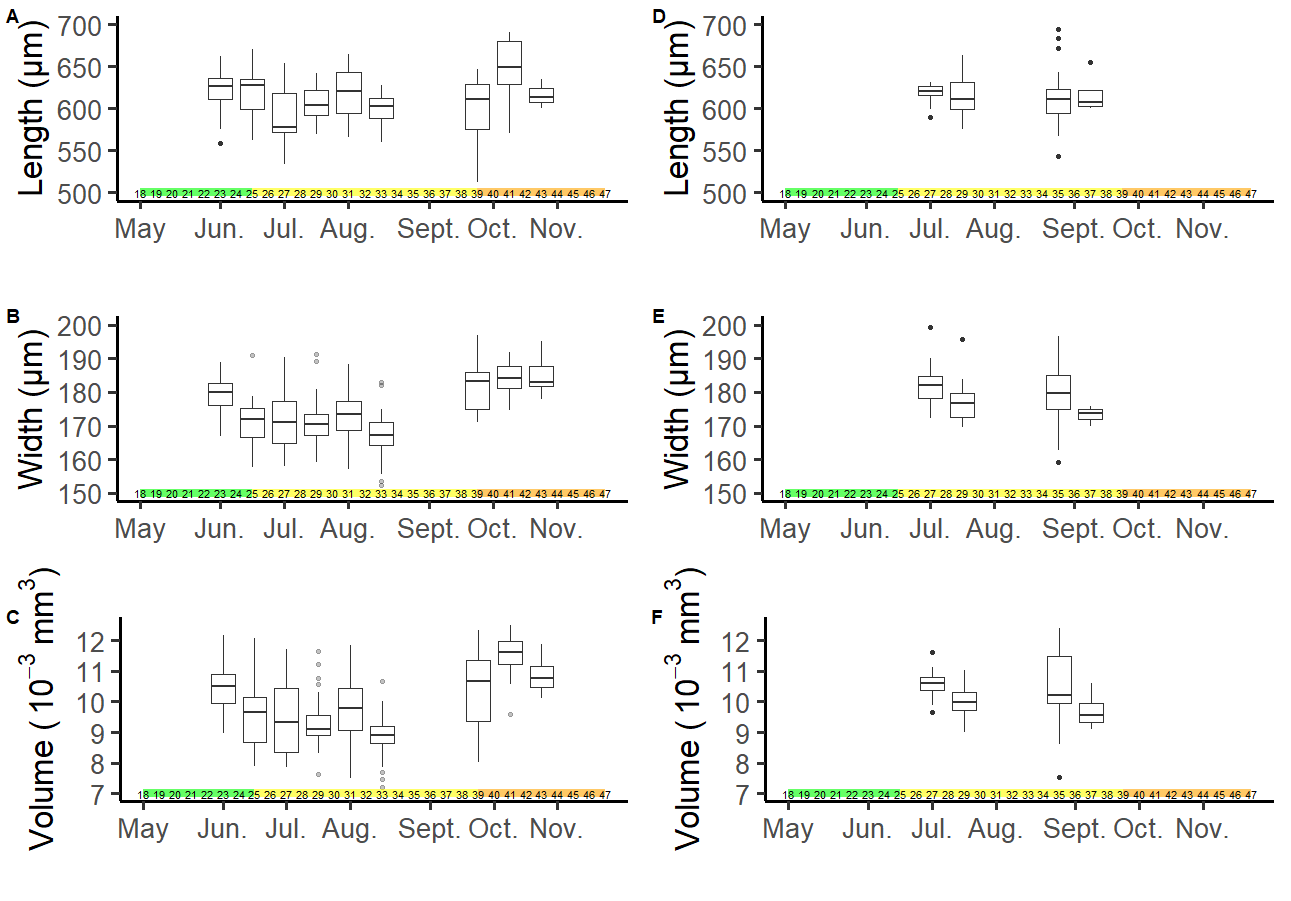


**Additional file 2. Figure 2. Changes in morphological parameters of *Ae. japonicus* eggs throughout the year.** Fig.4A Length; Fig.4B Width; Fig.4C Volume, data of 2019. Fig.4D Length; Fig.4E Width; Fig.4E Volume, data for 2020. Data originate from the Bas-Rhin region. Eggs are batched according to the calendar week of collection. Corresponding months and seasons are also shown in the coloured horizontal bars (green=spring, yellow=summer, orange=autumn). Data for week 37 in 2019 are discarded.

**Additional file 2. Table 1. Details of morphological metrics measured on *Ae. japonicus* eggs.** Data originate from the Bas-Rhin region. Length and width are measured 3 times per egg.

| 2019 | Length (µm) | Width (µm) | Volume (×10^-3^ mm^3^) |
| --- | --- | --- | --- |
| Minimum | 513 | 152 | 6.8 |
| Maximum | 691 | 197 | 12.7 |
| Mean | 616 | 176 | 10.0 |
| Standard-deviation | 29 | 8 | 1.2 |
| First quartile | 595 | 170 | 9.1 |
| Median | 619 | 175 | 10.0 |
| Third quartile | 635 | 182 | 10.8 |
| 2020 | Length (µm) | Width (µm) | Volume (×10^-3^ mm^3^) |
| Minimum | 543 | 159 | 7.5 |
| Maximum | 695 | 199 | 13.3 |
| Mean | 616 | 179 | 10.4 |
| Standard-deviation | 26 | 8 | 1.1 |
| First quartile | 601 | 175 | 9.8 |
| Median | 613 | 178 | 10.3 |
| Third quartile | 628 | 184 | 10.8 |

**Additional file 2 – Table 2. Sample size for morphological measurements, hatching success rate, mortality rate and diapause incidence.** Data come from the Bas-Rhin region.

| 2019 | Number of eggs | | | |
| --- | --- | --- | --- | --- |
|  | Collected | Measured | Tested after hatching | Bleached |
| Week |  |  |  |  |
| 23 | 395 | 62 | 64 | 19 |
| 25 | 858 | 33 | 15 | 1 |
| 27 | 247 | 15 | 18 | 4 |
| 29 | 943 | 40 | 61 | 29 |
| 31 | 833 | 83 | 55 | 6 |
| 33 | 1,078 | 33 | 30 | 14 |
| 35 | 382 | 0 | 13 | 13 |
| 37 | 19 | 8 | 9 | 5 |
| 39 | 34 | 33 | 29 | 25 |
| 41 | 152 | 28 | 10 | 10 |
| 43 | 215 | 16 | 15 | 15 |
|  |  | | | |
| 2020 | Number of eggs | | | |
| Week  25  27  29  31  33  35  37  39  41  43 | Collected  306  680  2,604  1,512  1,073  1,499  487  228  221  0 | Measured  0  0  14  19  0  0  25  4  0  0 | Tested after hatching  0  0  27  38  0  0  20  2  0  0 | Bleached  0  1  33  0  0  5  1  0  0  0 |
